# Silicon-Based Avalanche Photodiodes: Advancements and Applications in Medical Imaging

**DOI:** 10.3390/nano13233078

**Published:** 2023-12-04

**Authors:** Kirill A. Lozovoy, Rahaf M. H. Douhan, Vladimir V. Dirko, Hazem Deeb, Kristina I. Khomyakova, Olzhas I. Kukenov, Arseniy S. Sokolov, Nataliya Yu. Akimenko, Andrey P. Kokhanenko

**Affiliations:** 1 Department of Quantum Electronics and Photonics, Faculty of Radiophysics, National Research Tomsk State University, Lenin Av. 36, 634050 Tomsk, Russia; rahaf.douhan@gmail.com (R.M.H.D.); vovenmir@gmail.com (V.V.D.); deeb.hazem.syr@gmail.com (H.D.); homiackowa.kristina@yandex.ru (K.I.K.); okukenov@mail.ru (O.I.K.); ars856570@gmail.com (A.S.S.); kokh@mail.tsu.ru (A.P.K.); 2 Department of Engineering Systems and Technosphere Safety, Pacific National University, Tihookeanskaya St. 136, 680035 Khabarovsk, Russia; n_akimenko@inbox.ru

**Keywords:** photodetectors, medical imaging, positron emission tomography, single-photon emission computed tomography, time-of-flight positron emission tomography, computed tomography, fluorescence imaging, bioluminescence imaging, optical coherence tomography

## Abstract

Avalanche photodiodes have emerged as a promising technology with significant potential for various medical applications. This article presents an overview of the advancements and applications of avalanche photodiodes in the field of medical imaging. Avalanche photodiodes offer distinct advantages over traditional photodetectors, including a higher responsivity, faster response times, and superior signal-to-noise ratios. These characteristics make avalanche photodiodes particularly suitable for medical-imaging modalities that require a high detection efficiency, excellent timing resolution, and enhanced spatial resolution. This review explores the key features of avalanche photodiodes, discusses their applications in medical-imaging techniques, and highlights the challenges and future prospects in utilizing avalanche photodiodes for medical purposes. Special attention is paid to the recent progress in silicon-compatible avalanche photodiodes.

## 1. Introduction

Medical imaging plays a crucial role in modern healthcare by enabling the non-invasive visualization and characterization of anatomical structures, physiological processes, and the detection of diseases [1]. Advancements in medical-imaging technologies have revolutionized the field of diagnostics and therapy. One key component of medical-imaging systems is the photodetector, which is responsible for capturing and converting optical signals into electrical signals for further analysis and image reconstruction. Photodetectors play a critical role in detecting and capturing optical signals in various medical-imaging modalities [2].

Traditionally, photomultiplier tubes (PMTs) and photodiodes have been the primary photodetectors used in medical imaging. PMTs, known for their high sensitivity and fast response times, have been extensively employed in applications such as positron emission tomography (PET) and gamma cameras. Photodiodes, on the other hand, offer a compact size, low power consumption, and excellent linearity, making them suitable for applications such as pulse oximetry and optical coherence tomography (OCT). However, both of these traditional photodetectors have limitations that hinder their performance and restrict their applications in certain areas of medical imaging.

Photodetectors may have a limited sensitivity to low levels of light, which can be a challenge in situations where the incident light is weak. Additionally, their dynamic range, or the ability to capture a broad range of light intensities, might be constrained. Photodetectors can introduce noise into the signal, affecting the overall quality of the image. High noise levels can decrease the signal-to-noise ratio, making it challenging to distinguish the desired signal from background noise. Achieving a high spatial resolution is another crucial task in medical imaging to visualize fine details. The physical size of photodetectors and their pixel density can limit the spatial resolution of the imaging system. Additionally, the physical size of the detectors may influence the design and portability of imaging devices. The response time of photodetectors can impact the speed at which images are acquired. In some medical applications, especially those involving moving organs or dynamic processes, a slow response time may result in motion artifacts. One other problem is that some photodetectors are sensitive to changes in temperature, and variations in temperature can affect their performance. Finally, in X-ray imaging, photodetectors can be exposed to ionizing radiation, which may lead to radiation damage over time. This can affect the long-term performance and reliability of the detectors.

To overcome these limitations, there has been a growing interest in the use of avalanche photodiodes (APDs) for medical purposes [3]. APDs are semiconductor-based devices that operate based on the avalanche multiplication effect, which enables the detection of weak optical signals with high sensitivity [4]. This unique characteristic of APDs makes them well-suited for various medical-imaging modalities, including PET, single-photon emission computed tomography (SPECT), fluorescence imaging, and OCT.

APDs offer several key advantages, including a high responsivity, fast response times, enhanced stability, and compact size. These features make APDs highly attractive for medical-imaging applications, where sensitive detection, rapid signal acquisition, and miniaturization are essential requirements [5,6]. Additionally, APDs have demonstrated a superior performance in terms of quantum efficiency, timing resolution, and spatial resolution, further enhancing their suitability for advanced medical-imaging techniques [7].

In recent years, significant advancements have been made in APD technology to address the specific requirements of medical imaging. These advancements include improved quantum efficiency and responsivity, enhanced timing resolution, spatial resolution enhancement, integration with hybrid imaging systems, and cost-effective scale-up [8]. These developments have opened up new possibilities for high-quality medical imaging with improved diagnostic accuracy, enhanced imaging speed, and expanded imaging capabilities.

APDs can be integrated into detector modules, such as silicon photomultipliers (SiPMs). SiPMs are solid-state single-photon-sensitive devices based on an array of single-photon avalanche diodes (SPADs), implemented on a common silicon substrate. The dimensions of each individual SPAD can vary within the range of 10 to 100 μm, achieving a density of up to 10,000 per square millimeter. Each SPAD within a SiPM operates in Geiger mode (APD above breakdown) and is interconnected with others through a readout scheme. SiPMs are often considered analog devices due to the parallel reading of cells. SiPMs have a photodetection efficiency and gain comparable with PMTs. Moreover, SiPMs are not sensitive to external magnetic fields, have compact dimensions, and operate at lower voltages, enabling an extremely compact, light, and robust mechanical design.

However, despite the promising potential of APDs in medical imaging, there are still challenges that need to be overcome for their widespread adoption. These challenges include optimizing device performance for specific imaging modalities, addressing issues related to noise, dark counts, and crosstalk, developing efficient packaging and integration strategies, and ensuring cost-effectiveness for large-scale implementation.

The introduction of APDs has opened up new possibilities in medical imaging, offering an improved performance and novel capabilities. This article aims to provide an in-depth understanding of APDs and their applications in medical imaging. We will explore the key features of APDs that make them suitable for medical imaging, compare them with traditional photodetectors, and examine the advancements and challenges associated with their use for medical purposes. Particularly, this review will be focused on state-of-the-art silicon-based avalanche photodiodes. Additionally, we will discuss future perspectives and potential directions for further research and development in the field.

## 2. Avalanche Photodiodes: Principles and Features

### 2.1. Operating Principles of Avalanche Photodiodes

APDs are advanced semiconductor devices that utilize the effect of the absorption of electromagnetic radiation to convert light into an electrical signal. APDs offer a high responsivity and fast response times, making them well-suited for various medical-imaging applications. APDs operate on the principle of avalanche multiplication, which allows for the detection of individual photons and conversion into measurable electrical signals. This section provides an overview of the principles and operation of APDs.

#### 2.1.1. Photodetection Mechanism

**Figure 1:**
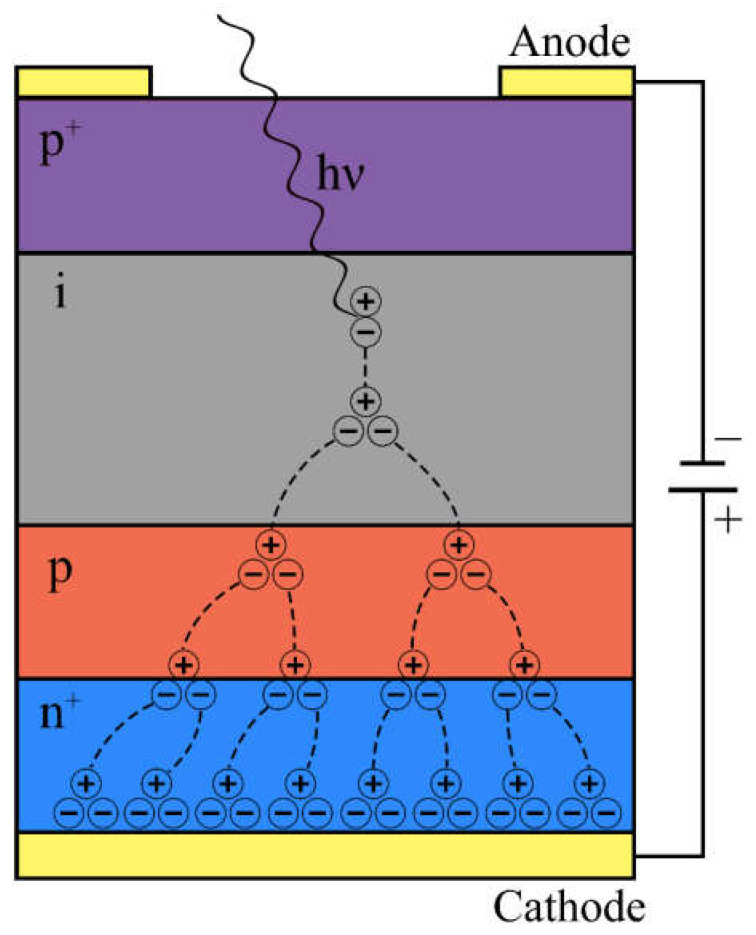
An avalanche photodiode and its operating principle.

The fundamental photodetection mechanism in APDs involves the absorption of photons by a semiconductor material, typically silicon, germanium, or III-V compound semiconductors. When a photon with energy greater than the bandgap of the material interacts with the semiconductor, it creates an electron-hole pair (Figure 1). The electron-hole pair is then accelerated by an electric field, typically applied through a reverse bias voltage, leading to the generation of additional electron-hole pairs through impact ionization. This phenomenon, known as the avalanche effect, results in a rapid multiplication of charge carriers, significantly amplifying the initial photo-generated signal. In essence, APDs serve as solid-state counterparts to photomultipliers.

The key distinction between an avalanche photodiode and a conventional photodiode lies in the internal signal amplification achieved through avalanche electron multiplication. In the layer structure of a conventional photodiode, typically with a p^+^–i–n^+^ configuration, an additional p-layer (p^+^–i–p–n^+^) is introduced in APDs (Figure 1).

Moreover, the dopant distribution profile is designed such that the highest electric field is concentrated in the region of the p-n junction. When the i-layer is illuminated, electron-hole pairs are generated. Due to the relatively weak field, the carriers migrate towards the respective terminals (anode or cathode). However, as free electrons from the i-layer enter the p-layer, their acceleration becomes more pronounced due to the intense electric field present. Within the conduction band of the p-layer, these accelerated electrons accumulate sufficient energy to excite other electrons from the valence band, leading to a phenomenon known as avalanche amplification or multiplication of the primary photocurrent [4].

#### 2.1.2. Avalanche Gain and Responsivity

The avalanche gain, defined as the ratio of the total number of generated charge carriers to the initial photo-generated carriers, determines the sensitivity and detection efficiency of APDs. When a photon flux with a density Φ (usually measured in cm^−2^) falls on the photodetector, the number of photons absorbed and converted into photoexcited charge carriers is ηΦ, where η is the quantum efficiency of the photodetector, showing the ratio of absorbed photons. Then, the photocurrent density (in amperes per cm^2^) of such a detector is determined by the photon flux density of the signal radiation, the quantum efficiency, and the avalanche gain. In other words, the avalanche gain is the number of charge carriers flowing in an electrical circuit for each absorbed signal photon. It has dimensionless units.

An important quantity characterizing the operation of photodetectors is their responsivity. Responsivity determines the value of response to an optical signal. The responsivity of a photodetector is defined as the magnitude of the photocurrent (amperes) at the output of the photodetector per unit of incident power (watts). Consequently, it is measured in A/W.

The avalanche gain and responsivity are influenced by several factors, including the applied bias voltage, the thickness of the depletion region, and the material properties of the semiconductor. By optimizing these parameters, APDs can achieve high gain values, allowing for the detection of weak optical signals.

#### 2.1.3. Noise Characteristics

APDs are subject to various noise sources that can affect their performance. The main noise sources include dark current noise, thermal noise, and excess noise due to avalanche multiplication. Dark current noise arises from the thermally generated carriers in the absence of light, leading to a baseline current level even when no optical signal is present. Thermal noise arises from the random thermal motion of charge carriers and can limit the signal-to-noise ratio of the detector. Excess noise due to avalanche multiplication is inherent to the avalanche process (in photomultiplier tubes, silicon solid-state photomultipliers, and APDs) and can affect the timing resolution and overall performance of the APD. The noise characteristics are usually characterized by the noise current density and are measured in A/cm^2^. Minimizing these noise sources is crucial for achieving high-quality and low-noise imaging with APDs.

#### 2.1.4. Timing Resolution

The timing resolution of APDs (in seconds) plays a critical role in medical-imaging applications that require precise time-of-flight measurements or coincidence detection. APDs offer fast response times in the order of picoseconds or nanoseconds, enabling accurate timing measurements. The timing resolution is influenced by various factors, including the transit time of charge carriers, the material properties, and the circuit design of the APD. Advanced timing techniques, such as time-correlated single-photon counting (TCSPC) and time-of-flight (TOF) measurements, can be employed to further improve the timing resolution and enable high-resolution imaging.

### 2.2. Key Features of Avalanche Photodiodes

APDs offer several features that make them highly suitable for various imaging applications. These features contribute to their superior performance and make them a preferred choice over traditional photodetectors. The key features of APDs include:

1. High responsivity. APDs are capable of detecting low-level optical signals with high responsivity due to the avalanche multiplication effect. The internal gain mechanism amplifies the initial photo-generated carriers, allowing for the detection of weak signals that would otherwise be challenging to detect with traditional detectors;
2. Fast response time. APDs exhibit fast response times, typically in the order of picoseconds or nanoseconds. This fast response enables the detection and measurement of rapid optical events, making APDs suitable for applications that require precise timing information or fast real-time imaging capabilities to capture dynamic processes in medical visualization;
3. Wide spectral range. APDs are available in various semiconductor materials, such as silicon, germanium, and III-V compounds, allowing for a wide spectral range of operation. This versatility enables APDs to be utilized across different wavelengths, including visible, near-infrared, and even X-ray regions, depending on the choice of the semiconductor material;
4. Hybridization and integration with imaging systems. APDs can be integrated with other imaging components to enhance their capabilities and enable hybrid imaging systems. For instance, the combination of APDs with scintillator crystals enables the development of PET detectors, where APDs detect the scintillation light produced by the interaction of positrons with the scintillator material. The compatibility of APDs with various imaging modalities, including PET, SPECT, and time-resolved imaging, allows for the integration of APDs into versatile and multimodal imaging systems.

Compared to traditional photodetectors, such as photomultiplier tubes and photodiodes, APDs offer distinct advantages that make them particularly suitable for medical-imaging applications. The following points may be highlighted as the advantages of APDs over traditional photodetectors:

1. Higher responsivity. APDs provide a higher responsivity compared to traditional photodetectors due to the avalanche multiplication effect. This allows for the detection of weaker optical signals and enhances the overall signal detection efficiency in medical-imaging systems;
2. Compact size. APDs are typically smaller in size and have a more compact form factor compared to traditional photodetectors. This compactness enables their integration into miniaturized imaging devices and facilitates the development of portable and handheld medical-imaging systems;
3. Lower voltage operation. APDs operate at lower bias voltages compared to PMTs, making them more energy-efficient and reducing the power requirements of the imaging system. This advantage is particularly beneficial for battery-powered or mobile medical-imaging applications;
4. Enhanced stability. APDs exhibit better long-term stability and reduced aging effects compared to PMTs. This stability ensures a consistent performance over time, reducing the need for frequent recalibration and maintenance in imaging systems.

In summary, APDs operate on the principle of avalanche multiplication and offer key features such as a high responsivity, fast response times, wide spectral range, and compact size, making them a superior choice over traditional photodetectors in medical-imaging applications. Understanding the principles and operation of APDs is crucial for optimizing their performance, minimizing noise sources, improving timing resolution, and integrating them with other imaging components. The next section will discuss the advancements and challenges associated with the use of APDs in various medical-imaging systems.

## 3. Applications of APDs in Medical Imaging

**Figure 2:**
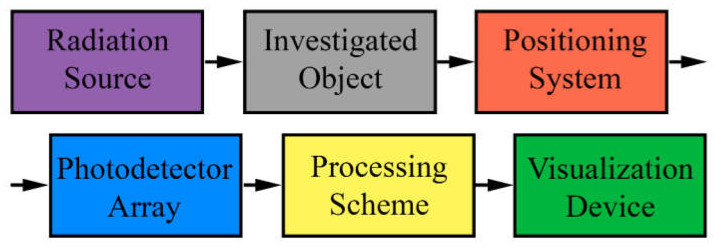
Scheme of the typical biomedical-imaging tool.

APDs have demonstrated significant potential in various medical-imaging applications. This section highlights the use of APDs in three key areas: positron emission tomography, optical imaging, and computed tomography. In all cases, an imaging installation consists of several main parts: source of radiation, positioning system, set of detectors, signal processing schemes, and visualization device (Figure 2). In this section we will consider the basics of various biomedical-imaging techniques and peculiarities of implementation of APDs in these tools.

### 3.1. Positron Emission Tomography (PET)

Positron emission tomography is a widely used imaging technique for studying physiological and biochemical processes, diagnosing and staging various diseases including cancer and neurological disorders. It is a non-invasive imaging modality that provides valuable information about organ function, metabolism, and molecular interactions [9]. PET imaging is based on the detection of positron-emitting radionuclides, which are short-lived radioactive isotopes. These isotopes are typically incorporated into biologically active molecules, such as glucose, water, or specific pharmaceuticals, to form radiotracers. The most commonly used radiotracer in PET is fluorodeoxyglucose (FDG), a glucose analog labeled with the positron-emitting isotope fluorine-18.

The imaging process begins with the administration of the radiotracer into the patient’s body, either by injection, inhalation, or ingestion, depending on the specific radiotracer and the organ or process being studied. Once inside the body, the radiotracer undergoes positron decay, wherein a positron (a positively charged particle) is emitted. Positrons have a short range in tissue, typically a few millimeters, before they encounter an electron. Upon encountering an electron, positrons undergo annihilation, resulting in the emission of two gamma photons traveling in approximately opposite directions. These photons are detected by a PET scanner, which consists of a ring of detectors surrounding the patient. The detectors in a PET scanner are typically scintillation crystals coupled to photodetectors, such as APDs or PMTs. When a gamma photon interacts with a scintillation crystal, it produces flashes of light, which are then converted into electrical signals by the photodetectors. The signals are processed and analyzed to reconstruct the distribution of radiotracer uptake in the body.

PET imaging provides three-dimensional information about the distribution of radiotracers in the body, allowing for the creation of detailed images called PET scans. These scans can reveal areas of abnormal or increased metabolic activity, indicating disease or dysfunction. PET is particularly useful in oncology, cardiology, neurology, and psychiatry, as it can assist in the early detection, staging, and evaluation of various diseases. One of the key advantages of PET is its ability to provide quantitative data. By measuring the concentration of radiotracers in different regions of interest, PET can provide valuable insights into the metabolic activity and other physiological processes occurring in the body. This quantitative information can be used for diagnostic purposes, treatment planning, and monitoring the response to therapy [10].

In recent years, PET technology has been further advanced with the development of hybrid imaging systems such as PET/CT (combined PET and computed tomography) and PET/MRI (combined PET and magnetic resonance imaging). These hybrid systems allow for the simultaneous acquisition of anatomical and functional information, enabling more accurate localization and characterization of abnormalities [11]. PET also continues to evolve with advancements in imaging technology and the development of novel radiotracers.

APDs have found extensive applications in PET due to their high quantum efficiency and excellent timing resolution at reasonable noise characteristics. In PET systems, APDs are commonly used as photodetectors in the scintillation crystals that detect the annihilation photons emitted by positron-emitting radiotracers. APDs offer enhanced responsivity and a high gain enabling the detection of low-energy photons and improving the overall image quality and quantitative accuracy. Additionally, APDs are particularly beneficial in time-of-flight PET, where the precise timing information of photon detection is crucial for reconstructing accurate images. Their fast response times enable precise time-of-flight measurements and better localization of the annihilation events, which can improve the spatial resolution and image reconstruction algorithms in PET. Silicon solid-state photomultipliers are now a mainstream solution for PET, enabling the creation of compact and highly sensitive PET systems [12,13,14].

### 3.2. Single-Photon Emission Computed Tomography (SPECT)

Single-photon emission computed tomography is a nuclear imaging technique used for functional imaging and molecular diagnostics. Like PET, SPECT relies on visualization of the distribution and function of radioactive tracers within the body [15]. It is commonly employed in clinical settings to diagnose and monitor a wide range of conditions, including cardiovascular diseases, neurological disorders, and cancer [16].

The SPECT imaging process begins with the injection of a radiopharmaceutical into the patient’s bloodstream. The radiopharmaceutical contains a gamma-emitting radioactive isotope, such as technetium-99m, which emits single photons during radioactive decay. The radiopharmaceutical is designed to selectively accumulate in specific organs or tissues of interest based on its biological properties. After the injection, the patient is positioned within a SPECT scanner, which consists of one or more gamma camera heads that rotate around the body. Each gamma camera head comprises a collimator, which is a lead or tungsten plate with small holes, and a scintillation crystal. The collimator allows only photons emitted in specific directions to pass through, while the scintillation crystal converts the gamma photons into visible light. As the gamma camera head rotates around the patient, it detects the emitted gamma photons that escape from the body and pass through the collimator. The scintillation crystal within the gamma camera head converts the detected photons into flashes of light. These light flashes are then detected by PMTs or solid-state photodetectors, which produce electrical signals proportional to the intensity of the light. The signals from the photodetectors are sent to a computer system, where sophisticated algorithms are employed to reconstruct the three-dimensional distribution of the radiotracer within the body. This reconstruction process generates cross-sectional slices, similar to those obtained in computed tomography (CT) scans, allowing for detailed visualization of the activity and function of the target organs or tissues.

One of the key advantages of SPECT is its ability to provide functional information, allowing the assessment of physiological processes in addition to anatomical structure. By using different radiopharmaceuticals with a specific affinity to various organs or processes, SPECT can assess blood flow, metabolism, receptor binding, and other functional parameters. SPECT offers several benefits in clinical practice. It is widely available, non-invasive, and relatively cost-effective compared to other imaging modalities. It provides valuable diagnostic information, helps in treatment planning, and aids in monitoring the response to therapy. SPECT is particularly useful in cardiology for assessing myocardial function, in neurology for evaluating cerebral blood flow and neuronal function, and in oncology for detecting and staging tumors.

However, SPECT also has some limitations. It has a lower spatial resolution compared to other imaging techniques like PET. The limited spatial resolution of SPECT can lead to reduced anatomical detail and decreased accuracy in localizing small lesions. Additionally, the imaging process in SPECT takes longer compared to PET, which can be a challenge for patients with limited mobility or discomfort.

Recently, advancements in SPECT technology have been made to address some of these limitations. Hybrid systems, such as SPECT/CT, combine the functional information from SPECT with the anatomical details provided by CT scans. Furthermore, the development of new collimator designs, advanced reconstruction algorithms, and higher-resolution detectors have contributed to enhanced image quality and spatial resolution in SPECT imaging.

APDs have been successfully employed in SPECT systems, providing enhanced responsivity, improved energy resolution, and reduced image blurring compared to conventional PMTs. The high gain of APDs enables the detection of weak gamma-ray signals, allowing for more accurate and detailed imaging. The integration of APDs into SPECT detectors has the potential to improve the image quality, increase the spatial resolution, and reduce the system size.

### 3.3. Time-of-Flight Positron Emission Tomography (TOF-PET)

Time-of-flight positron emission tomography is an advanced imaging technique that enhances the capabilities of conventional PET by incorporating time information into the imaging process. Unlike traditional PET, which relies solely on the detection of gamma photons and their spatial distribution, TOF-PET also utilizes the time-of-flight or time difference between the emission of a positron and the detection of the resulting annihilation photons to improve spatial resolution and signal-to-noise ratio (SNR) [17].

The principle behind TOF-PET is based on the fact that gamma photons travel at the speed of light. By accurately measuring the time it takes for the annihilation photons to reach the detectors after the emission of the positron, TOF-PET can provide additional information about the location of the annihilation event along the line of response (LOR).

The main advantage of TOF-PET is its ability to precisely determine the origin of annihilation photons, especially in cases where multiple annihilation events occur simultaneously along different LORs. By knowing the time difference between the photons’ emission and detection, the system can calculate the respective distances traveled by the photons and narrow down the possible locations of the annihilation event. This improved localization helps reduce image blurring and improves spatial resolution, leading to better lesion detectability and overall image quality.

Meanwhile, TOF-PET offers several other benefits over conventional PET imaging. First, it provides faster image acquisition times, as the temporal information allows for more efficient reconstruction algorithms. With shorter acquisition times, patients experience reduced scan durations, leading to increased patient comfort and improved workflow in clinical settings. Another advantage of TOF-PET is its superior signal-to-noise ratio. By accurately localizing the annihilation events, TOF-PET reduces the number of random coincidences, which occur when two unrelated photons are erroneously detected as simultaneous events. Moreover, TOF-PET enables lower administered radiotracer doses while maintaining image quality. With accurate timing information, TOF-PET compensates for the reduced statistics due to lower activity levels, making it suitable for pediatric and oncology applications where minimizing radiation exposure is important [18].

Despite its advantages, TOF-PET also presents some challenges. One significant challenge is the accurate timing resolution required for precise time measurements. The timing resolution determines the accuracy of the calculated time-of-flight information and is influenced by various factors, such as the detector technology, electronics, and the properties of the scintillation crystals used. Additionally, TOF-PET systems are more complex and expensive compared to conventional PET systems due to the need for fast and precise timing measurements. The detectors and electronics must be capable of high timing resolution to achieve the desired image quality. However, advancements in detector technology, such as the development of fast scintillators and dedicated time-resolving photodetectors like SiPMs, have facilitated the implementation of TOF-PET in clinical practice [19].

APDs with their fast response times and excellent timing resolution are well-suited for TOF-PET systems [20]. By accurately measuring the time-of-flight information, APDs enable precise localization of the annihilation events and reduce image artifacts. TOF-PET systems incorporating APDs have shown superior image quality and enhanced lesion detectability. The combination of APDs with TOF-PET has the potential to enhance the diagnostic accuracy and quantification capabilities of this imaging modality.

### 3.4. Computed Tomography (CT)

Computed tomography is a widely used imaging technique for anatomical visualization and disease diagnosis. CT uses X-rays and computer processing to create detailed cross-sectional images of the body. It provides a three-dimensional view of internal structures, allowing for the diagnosis, treatment planning, and monitoring of various medical conditions.

The CT imaging process involves the use of a specialized X-ray machine called a CT scanner. The patient lies on a motorized table that moves through the center of the scanner, while the X-ray tube and detector array rotate around them. The X-ray tube emits a narrow beam of X-rays that pass through the body and are detected by the detector array on the opposite side. The detectors measure the intensity of the X-rays that pass through the body, which varies depending on the density of the tissues encountered. The data from the detectors are then sent to a computer, which reconstructs the information into cross-sectional images or slices. These slices can be further processed to create three-dimensional images of the scanned area.

One of the key features of CT is its ability to differentiate tissues based on their X-ray attenuation properties. Different tissues, such as bone, muscle, and organs, have different densities and X-ray absorption characteristics. This enables the generation of high-resolution images that provide detailed anatomical information. Modern CT scanners can acquire images in rapid succession, allowing for the dynamic imaging of structures and the visualization of organ function over time. This capability is particularly useful in cardiac imaging, where CT angiography can assess blood flow and detect blockages in the coronary arteries. In some cases, contrast agents may be used to enhance the visibility of certain structures or abnormalities. These contrast agents, typically iodine-based, are administered orally, intravenously, or rectally, depending on the area being imaged. They help to highlight blood vessels, tumors, or other areas of interest, aiding in the diagnosis and characterization of various conditions.

CT imaging is used in a wide range of medical specialties, including radiology, oncology, neurology, orthopedics, and emergency medicine. It is valuable for diagnosing conditions such as fractures, tumors, infections, and vascular diseases. CT scans are also commonly used for surgical planning and image-guided interventions.

While CT imaging provides detailed anatomical information, it does involve exposure to ionizing radiation. However, modern CT scanners are equipped with dose-reduction techniques to minimize radiation exposure while maintaining image quality. It is important for healthcare professionals to weigh the benefits of CT imaging against the potential risks and ensure that the procedure is justified for each patient.

APDs have emerged as a promising alternative to traditional PMTs in CT systems, offering advantages such as a higher spatial resolution, compact size, and lower power consumption. The use of APDs in CT detectors enables improved image quality, faster acquisition times, and a reduced radiation dose to the patient. SiPMs, a type of APD, have gained attention in CT due to their high photon detection efficiency, excellent timing resolution, and immunity to magnetic fields [7]. SiPM-based CT detectors have shown promise in applications such as cardiovascular imaging and CT angiography. APDs can be integrated into hybrid CT systems, such as PET/CT and SPECT/CT, enabling the combination of anatomical and functional information for more comprehensive diagnostics [11].

### 3.5. Fluorescence Imaging

In addition to tomography methods, APDs have been employed in optical imaging techniques, such as fluorescence molecular imaging and bioluminescence imaging. These techniques rely on the detection of light emitted from fluorescent probes or bioluminescent reporters to visualize molecular and cellular processes [21,22].

Fluorescence imaging is a non-invasive medical-imaging technique that utilizes the emission of fluorescent light from molecules or probes to visualize specific biological processes, structures, or targets within living organisms. It has gained significant popularity in biomedical research and clinical applications due to its high sensitivity, real-time imaging capabilities, and ability to provide molecular-level information.

The fluorescence imaging process involves the use of fluorescent probes or dyes that are designed to bind to specific molecules or structures of interest. These probes contain a fluorophore, a molecule that can absorb light at a specific wavelength and emit light at a longer wavelength upon excitation. The excitation light, typically provided by a light source such as a laser or LED, is directed onto the tissue or sample being imaged. When the excitation light interacts with the fluorescent probe, the fluorophore molecules become excited and subsequently emit fluorescent light at a longer wavelength. This emitted light is then captured by a detector, such as a camera or a specialized imaging system, which converts the light signals into a visual image or quantitative data [23].

One of the key advantages of fluorescence imaging is its high sensitivity and specificity [24]. By using fluorescent probes that selectively bind to specific molecules or targets, researchers and clinicians can visualize and track the distribution, localization, and activity of these targets within biological samples or living organisms. This allows for the study of various cellular processes, such as gene expression, protein interactions, enzyme activity, and molecular signaling pathways.

Fluorescence imaging can also be combined with other imaging modalities to provide complementary information. For example, combining fluorescence imaging with anatomical imaging techniques like CT or MRI allows for the correlation of molecular information with structural context, enhancing the understanding and interpretation of biological processes.

In recent years, there have been significant advancements in fluorescence imaging technology. These include the development of advanced fluorescent probes with improved brightness, photostability, and targeting capabilities. Additionally, the emergence of multiplexing techniques enables the simultaneous imaging of multiple targets using different fluorophores, providing a more comprehensive view of complex biological systems.

Fluorescence imaging has found widespread applications in various fields, including cancer research, neurobiology, immunology, and drug discovery. It has contributed to the understanding of disease mechanisms, the development of new therapeutic approaches, and the evaluation of treatment responses. Moreover, fluorescence imaging has the potential for clinical translation, with applications in surgical guidance, tumor detection, and the monitoring of therapeutic interventions.

APDs have been employed in fluorescence imaging systems to enhance sensitivity and improve signal detection [25,26]. The high gain of APDs enables the detection of weak fluorescence signals, allowing for the visualization of subtle molecular events. APDs can be utilized in various fluorescence imaging modalities, including fluorescence microscopy, fluorescence lifetime imaging microscopy (FLIM), and fluorescence molecular tomography (FMT). For all these techniques, APDs offer advantages in terms of their high responsivity and wide dynamic range, enabling the detection of weak light signals and facilitating quantitative measurements.

### 3.6. Bioluminescence Imaging

Bioluminescence imaging is a non-invasive imaging technique that utilizes the light emitted by bioluminescent organisms or genetically modified cells to visualize biological processes in living organisms. It relies on the production of light by naturally occurring or engineered bioluminescent molecules, such as luciferase enzymes, which emit photons upon a specific biochemical reaction [27]. The process of bioluminescence imaging involves introducing a bioluminescent probe or gene into the target cells or organisms of interest. This probe or gene encodes for a bioluminescent protein which can produce light through a series of enzymatic reactions.

To initiate the bioluminescent reaction, a substrate molecule, such as luciferin, is administered to the subject. When the substrate interacts with the bioluminescent protein, it undergoes an enzymatic reaction that results in the emission of light. The emitted photons are then detected by a highly sensitive camera or imaging system, allowing for the visualization and quantification of the bioluminescent signal.

One of the main advantages of bioluminescence imaging is its high sensitivity [28]. The emitted bioluminescent signal is relatively weak, but it can be detected with very low background noise due to the absence of endogenous bioluminescence in mammalian tissues. This enables the detection of even small numbers of bioluminescent cells or organisms in vivo. Bioluminescence imaging is commonly used in preclinical research to study various biological processes and phenomena. It has been extensively employed in fields such as cancer research, immunology, neurobiology, and infectious disease studies. By introducing bioluminescent reporter genes into specific cell types or organisms, researchers can track and monitor their behavior, migration, proliferation, and response to stimuli or treatments over time [29].

Another significant advantage of bioluminescence imaging is its ability to provide longitudinal and real-time information. Since the emitted light is directly proportional to the number of bioluminescent cells or the activity of the bioluminescent reporter, changes in signal intensity can be correlated with the underlying biological processes. This allows for the assessment of disease progression, therapeutic responses, and the evaluation of novel therapies in living subjects over time.

However, it is important to note that bioluminescence imaging has limitations. The emitted light is subject to scattering and absorption within tissues, which can limit the spatial resolution and depth penetration of the imaging signal. Additionally, the emitted photons can only provide information about the location and intensity of the bioluminescent signal, without revealing detailed anatomical structures or functional parameters. To overcome some of these limitations, bioluminescence imaging is often combined with other imaging modalities, such as CT or MRI. This allows for the integration of anatomical information with the bioluminescent signal, providing a more comprehensive view of the target site and facilitating data interpretation [30].

APDs offer a high responsivity and the detection of low signals, making them well-suited for bioluminescence imaging applications. By incorporating APDs into bioluminescence imaging systems, researchers can achieve improved signal detection, higher spatial resolution, and enhanced quantification of bioluminescent signals [31]. Their compact size and compatibility with small-animal imaging systems make APDs suitable for preclinical research and drug development studies.

### 3.7. Optical Coherence Tomography (OCT)

Furthermore, APDs can be integrated with advanced imaging modalities like optical coherence tomography to enhance imaging capabilities. Optical coherence tomography is a non-invasive imaging technique that provides cellular level cross-sectional imaging of biological tissues in real time. It utilizes low-coherence interferometry to capture and analyze the backscattered or back-reflected light from biological tissues [32].

The principle of OCT is analogous to ultrasound imaging, but instead of sound waves, it employs near-infrared light. The system consists of a broadband light source, typically a superluminescent diode or a femtosecond laser, which emits light with a broad spectrum of wavelengths. The light is split into two paths: the sample arm and the reference arm. In the sample arm, the light is directed towards the tissue being imaged. A scanning mechanism, such as a galvanometer mirror, is used to steer the light beam across the tissue surface or within the tissue. As the light interacts with the tissue, a portion of it is backscattered or back-reflected due to variations in the refractive index and scattering properties of different tissue structures. Simultaneously, in the reference arm, the light travels through a reference path of known length. This path usually consists of a mirror or a reference reflector. The light from the reference arm is combined with the backscattered light from the sample arm using a beamsplitter. The combined light from both arms is then directed to an interferometer, where interference occurs between the reference light and the backscattered light from the sample. The interference pattern carries information about the depth and intensity of the backscattered light at different locations within the tissue.

To obtain cross-sectional images, the interference pattern is detected using a photodetector and processed by an OCT system. The system measures the time delay or phase difference between the reference and sample arms at each depth position. By scanning the light beam across the tissue or by moving the sample, a series of depth profiles are acquired. By combining multiple depth profiles obtained at adjacent positions, a two-dimensional cross-sectional image is generated. These two-dimensional images can be further combined to form three-dimensional volumetric images of the tissue [33].

OCT provides high-resolution images with a micrometer-scale axial and lateral resolution, allowing for the detailed visualization of tissue structures and cellular morphology. It has a wide range of applications in ophthalmology, dermatology, cardiology, gastroenterology, and other medical fields. One of the significant advantages of OCT is its non-invasive nature, which enables the real-time imaging of biological tissues without the need for tissue excision or contrast agents. It allows clinicians and researchers to observe tissue morphology, identify abnormalities, and monitor disease progression or treatment response. Moreover, OCT can be enhanced with various imaging modes to extract additional information. For example, Doppler OCT can measure blood flow velocity in vessels, polarization-sensitive OCT can assess tissue birefringence and polarization properties, and spectroscopic OCT can analyze tissue composition and identify specific biomarkers [34].

APDs have been employed in OCT systems to enhance detection sensitivity and improve image quality [35]. The high gain characteristics of APDs enable the detection of weak optical signals, allowing for deeper tissue penetration and higher imaging depths [36]. By utilizing APDs in OCT, one can achieve a higher resolution, faster imaging speeds, and improved visualization of structural and functional information in biological tissues [37]. High-resolution imaging and improved depth penetration for applications such as ophthalmology, cardiovascular imaging, and cancer detection have already been obtained.

Overall, APDs have demonstrated their potential in various medical-imaging applications, including PET, optical imaging, and CT. Their unique characteristics such as a high responsivity and fast timing response contribute to enhanced image quality, improved diagnostic accuracy, and reduced radiation exposure. Continued advancements in APD technology and their integration into imaging systems hold great promise for further advancements in medical imaging.

## 4. Progress in Silicon-Based Avalanche Photodiodes

Silicon-compatible avalanche photodiodes (including single-photon avalanche diodes) based on group IV materials, such as silicon and germanium, and fabricated using standard CMOS technology, hold great promise for biomedical applications [38]. Compared to III-V devices, silicon/germanium APDs and SPADs exhibit reduced afterpulsing effects and offer a cost-effective device platform [39,40,41]. Researchers are currently focused on improving these devices by addressing issues related to noise, cost, and compatibility with traditional circuits, in order to fully leverage the advantages of silicon-based systems [42,43,44,45].

A crucial component of modern APDs is the guard ring, which plays a significant role in preventing premature edge breakdown and optimizing the electric field distribution in the multiplication region [46,47,48]. The selection of appropriate physical dimensions, particularly the active radius and guard ring size, is essential for achieving a superior performance in silicon APDs.

**Figure 3:**
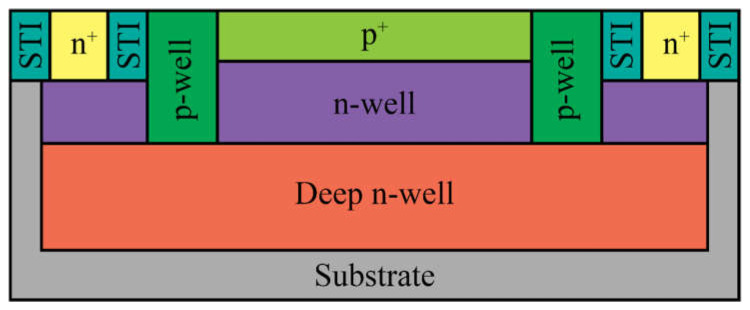
Cross-section of the APD with a guard ring.

In 2015, Malass et al. [49] presented the performance characteristics of SPADs fabricated using a standard 180 nm CMOS process. The active area was based on a p^+^/n-well junction within a deep n-well on a high-resistivity p-substrate (Figure 3). To prevent premature breakdown, a double p-well/STI (shallow trench isolation) guard ring was incorporated. The low-doped p-well guard ring was added to isolate the multiplication region from the STI implant, reducing high dark noise caused by faulty detections triggered by carrier injections from the STI interface into the multiplication area.

In 2018, Accarino et al. [50] showed a square-shaped photo-carrier diffusion SPAD with a narrow depletion region and a wide photo collection region. The design of the guard ring effectively reduced the electric field and protected the active junction. The width of the guard ring and its distance from the n-well were carefully chosen to minimize the electric field at the p-n interface, which made it possible to achieve an outstanding performance with this SPAD design.

Later in 2018, Wang et al. [51] proposed an APD with the multiplication region surrounded by a virtual guard ring. A gradient doping profile is applied to the central implantation area, where the doping concentration increases from the surface to deeper into the wafer. This retrograde doping profile enhances the electric field in the central anode active region and serves as a virtual guard ring. This design helped to achieve a high photodetection efficiency in the spectral range of 650 nm to 950 nm.

In 2019, Shin et al. [45] presented a silicon SPAD with a deep p-substrate virtual guard ring fabricated using a deep well diffusion process via a 110 nm CMOS technology. As a deeper multiplication region requires a deeper guard ring, separate layers were doped and connected through a diffusion process to create the virtual guard ring. Shallow trench isolation layers were added between the source and drain layers to prevent the formation of a high electric field between the anode and cathode. The deep virtual guard ring surrounds the multiplication area, preventing electric field leakage at the edge of the multiplication region while maintaining a low doping concentration of the p-substrate compared to the conventional guard ring doping. The authors also investigated the effect of the physical variation in the active radius and the width of the guard ring on the device characteristics.

Recent advances in molecular beam epitaxy (MBE) have enabled the effective and reliable fabrication of germanium-on-silicon APDs, showcasing impressive performance characteristics [42,52,53,54,55,56,57]. In these detectors, light absorption occurs in the germanium layer, while the multiplication process takes place in silicon. MBE of germanium on a silicon substrate has emerged as the most promising fabrication method for these structures. This technology has allowed the achievement of operating characteristics in germanium/silicon detectors that are comparable to those of competing A3B5 devices [58].

**Figure 4:**
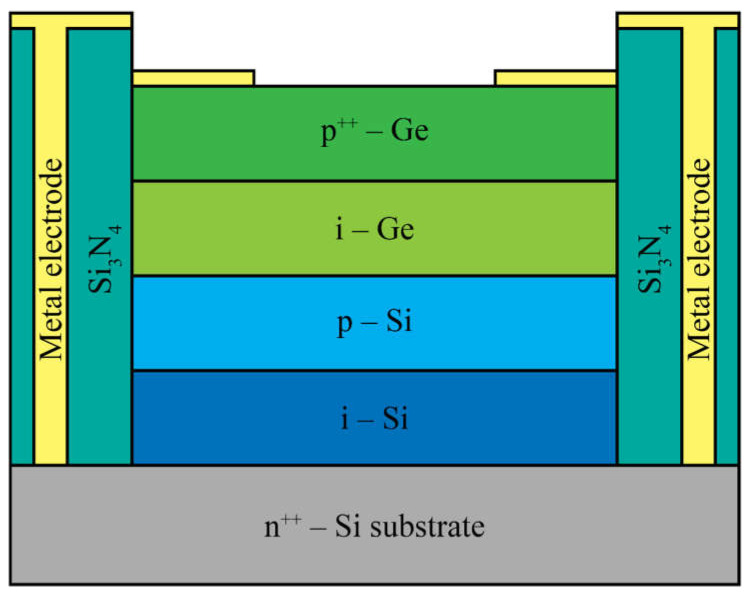
Cross-section of the multilayer germanium-on-silicon APD.

The first demonstration of a single-photon detector utilizing a germanium absorber coupled with a silicon multiplication region was conducted in 2002 by Loudon et al. [59]. They showed an improvement in detection efficiency at a wavelength of 1210 nm compared to pure silicon structures, utilizing SiGe/Si multiple quantum wells. In 2011, Lu et al. [60] reported a germanium-on-silicon SPAD at the wavelength of 1310 nm. In 2013, Warburton et al. [61] proposed multilayer germanium-on-silicon structures for APDs (Figure 4). They reported, for the first time, measurements of detection efficiency at the wavelength of 1550 nm using a germanium-on-silicon avalanche diode detector.

**Figure 5:**
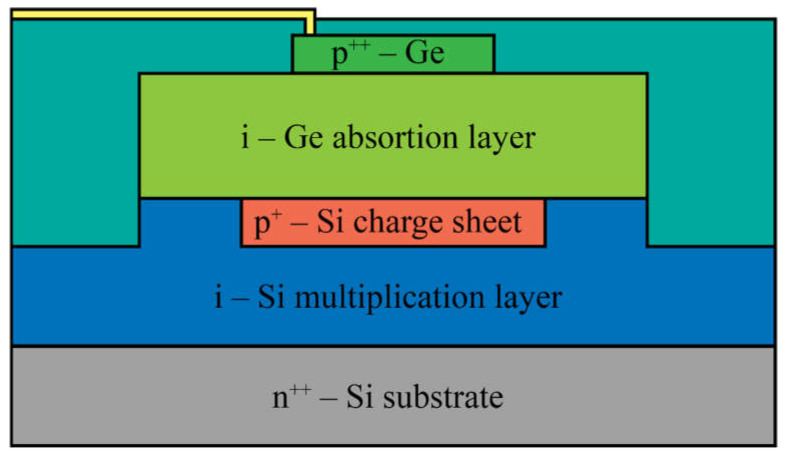
Cross-section of the separate absorption, charge, and multiplication (SACM) planar germanium-on-silicon SPAD.

Vines et al. [62] have recently introduced a new generation of group IV devices for single-photon detection in the near-infrared (NIR) and short-wave infrared (SWIR) regions. They present the first normal-incidence, planar germanium-on-silicon SPAD with high-performance single-photon operation, showcasing high-efficiency detection and low afterpulsing effects. The devices described in the paper utilized the separate absorption, charge, and multiplication (SACM) structure (Figure 5).

The incident SWIR radiation was absorbed in the germanium absorption region, while the signal amplification occurred in the silicon multiplication region. A selectively implanted charge sheet was placed between these regions to control the electric field, ensuring a sufficiently high field in the multiplication region for avalanche breakdown and a low field in the absorption region to prevent band-to-band and trap-assisted tunneling. The germanium layer maintained a modest electric field to facilitate the efficient drift of photogenerated electrons into the multiplication region [62]. The same authors also achieved a high single-photon detection efficiency at 1310 nm using a planar geometry germanium-on-silicon SPAD [63]. Thorburn et al. [42] recently utilized the same geometry as Vines et al. [62] to develop highly efficient planar germanium-on-silicon SPADs with the spectral range extended into the SWIR region. These results highlight the prospects of germanium-on-silicon SPADs operating at near-room temperature.

Another design approach for germanium-on-silicon avalanche detectors incorporates a waveguide structure [64,65,66,67,68]. The use of such structures makes it possible to significantly increase the effective absorption length of radiation that results in multiplying the sensitivity of the receiver [41].

Efforts are also being directed toward the development of APDs with silicon/germanium nanostructures. The results of these studies suggest that future nanostructured APDs may surpass traditional ones in terms of efficiency and frequency parameters [69,70,71,72,73].

For instance, Stepina et al. [69] demonstrated the possibility of using germanium quantum dots on silicon and silicon-on-insulator substrates for single-photon detection. Similarly, Ali and Richardson [70] fabricated p-i-n photodetectors with silicon–germanium superlattices that exhibited absorption up to 1770 nm, which can be utilized as absorption regions for silicon-based avalanche photodiodes.

Ma et al. [71] proposed a nanostructured silicon SPAD based on a thin film APD with nanocone gratings coated on both sides. The top silicon nitride nanocone grating served as a broadband antireflection coating, while the bottom silicon nanocone grating aimed to strongly scatter light toward the lateral direction, with its parameters adjusted for maximum absorption. Optical and electrical simulations showed a significant performance enhancement compared to conventional silicon SPAD devices.

Another approach for creating nanostructured APDs involves photon trapping structures [6]. For example, Zang et al. [72] proposed for these structures methods of lithography followed by etching. They demonstrated a light-trapping, thin-junction silicon single-photon avalanche diode that diffracted incident photons into the horizontal waveguide mode, significantly increasing the absorption length. This nanostructured SPAD exhibited improved near-infrared detection efficiency.

Researchers are also considering the use of other group IV materials, primarily tin, for the creation of APDs and SPADs [44,66,74]. For example, Zhang et al. [74] demonstrated a mesa-type normal incidence SACM GeSn/Si APD with a high responsivity and low dark current at wavelengths of 1310 nm and 1550 nm. Additionally, Soref et al. [66] reported on the design and simulation of waveguide-based GeSn/Si SPADs for a wavelength of 1550 nm. They showed that the GeSn/Si system could be a promising candidate for realizing SPADs operating at room temperature for integrated quantum photonics. However, these investigations are still in the early stages [44].

Today, there are many manufacturers of commercially available APDs, SPADs, and SiPMs, for example, Hamamatsu [75], OSI Optoelectronics [76], Ushio [77], AMS Technologies [78], Excelitas [79], Micro Photon Devices [80], Onsemi [81], Sony Semiconductor Solutions Group [82], STMicroelectronics [83], and Advanced Photonix [84] to name but a few. They offer a wide range of discrete detectors and multi-element detector arrays for all sorts of biomedical-imaging applications, operating from ultraviolet to near infrared spectral diapasons, and optimized for various tasks such as low-level light detection, time-of-flight measurements, high-speed response, or wavelength-optimized operation. To choose the right detector for the desirable biomedical visualization tool, one should, first of all, outline the spectral range of sensitivity, and then decide whether the maximum responsivity or enhanced timing resolution are crucial for the task being solved, because these two characteristics cannot be improved simultaneously.

All of the silicon APDs presented in this review, designed using standard CMOS technology, offer researchers the advantage of easy integration with well-established conventional technology [85,86]. These detectors demonstrate excellent operating characteristics and cost-effectiveness. The reviewed results illustrate the clear potential for integrating group IV materials APDs and SPADs with silicon CMOS technology for low-cost, high data rate detector array imaging in the visible, NIR, and SWIR bands [87,88,89]. These advancements are crucial for emerging biomedical applications.

## 5. Advancements and Challenges

APDs have demonstrated their potential to enhance various medical-imaging techniques, providing improved detection capabilities. The ongoing advancements in APD technology offer exciting prospects for the future of medical imaging. However, several challenges need to be addressed, including scale-up for larger detector arrays, cost reduction, and further improvements in quantum efficiency and timing resolution.

Improved quantum efficiency and responsivity. One of the key advancements in APDs for medical imaging is the improvement in quantum efficiency and responsivity. Efforts have been made to enhance the APD’s ability to detect and convert incoming photons into measurable electrical signals [90]. This includes optimizing the material properties and structure of the APD, as well as incorporating advanced photon management techniques [91]. By increasing the quantum efficiency and responsivity of APDs, the overall performance of medical imaging systems can be significantly improved, leading to better image quality and increased diagnostic accuracy.

Timing resolution enhancement. Timing resolution is a critical parameter in medical-imaging applications, especially in PET and TOF-PET [92]. APDs have shown remarkable advancements in timing resolution, enabling precise measurements of the arrival time of photons [93]. This enhancement in timing resolution allows for more accurate localization of detected events, leading to improved spatial resolution and image quality. Ongoing research aims to further improve the timing resolution of APDs, pushing the limits of temporal accuracy in medical-imaging systems.

Spatial resolution enhancement. Spatial resolution plays a vital role in medical imaging, as it determines the ability to distinguish small structures and accurately localize abnormalities [94]. APDs have demonstrated advancements in spatial resolution by reducing the size of individual detection elements and minimizing crosstalk between neighboring elements. These improvements enable finer spatial sampling and a better delineation of features in the acquired images. Additionally, advancements in APD packaging and readout electronics contribute to minimizing noise and improving the overall spatial resolution performance [95,96].

Integration with hybrid imaging systems. Hybrid imaging systems, combining multiple imaging modalities, have gained significant attention in medical imaging [11,97]. APDs offer compatibility and integration capabilities with other imaging technologies, such as MRI and CT [98,99]. The unique properties of APDs, such as their compact size, low power consumption, and immunity to magnetic fields, make them suitable for hybrid imaging systems. This integration allows for simultaneous data acquisition from different modalities, providing complementary information and improving the diagnostic capabilities of medical imaging.

Cost and scale-up challenges. While APDs offer significant advancements in medical imaging, there are challenges related to cost and scale-up for widespread adoption [100]. Currently, the cost per unit area of APDs needs to be reduced to be comparable to conventional photodetectors, such as PMTs. Additionally, scaling up the APD technology for large-area detectors, such as those used in whole-body PET scanners, presents manufacturing and assembly challenges. Efficient packaging and integration techniques are required to accommodate a higher number of APD pixels and ensure reliable performance in commercial medical-imaging systems [101].

Addressing these challenges will be crucial for the broader implementation of APDs in medical imaging, allowing for improved patient care, more accurate diagnoses, and advancements in medical research.

## 6. Future Perspectives

APDs have already demonstrated their potential in various medical-imaging applications. As the field continues to evolve, there are several exciting future perspectives that can further enhance the capabilities of APDs and drive advancements in medical imaging.

Enhanced performance in low-light conditions. One of the key areas of focus for future APD development is the improvement of performance in low-light conditions [102]. Medical imaging often involves imaging scenarios with a low photon flux, such as deep-tissue imaging or imaging with radiotracers in small concentrations. APDs with enhanced responsivity and lower noise characteristics will enable the better detection of weak signals, leading to improved image quality and enhanced diagnostic accuracy. Ongoing research efforts aim to optimize the APD design, material properties, and signal-processing techniques to achieve a higher signal-to-noise ratios and maximize the detection efficiency in low-light conditions [103].

Multispectral imaging capabilities. The integration of multispectral imaging capabilities into APDs holds great promise for future medical-imaging applications. By incorporating wavelength-selective materials or filters into the APD structure, it becomes possible to detect and differentiate photons of different wavelengths. This opens up new opportunities for functional imaging, such as spectroscopic imaging or targeted imaging with specific fluorophores or contrast agents. Multispectral APDs can provide valuable information about tissue composition, molecular interactions, and physiological processes, enabling more comprehensive and personalized diagnoses [104,105,106].

Development of compact and portable systems. Advancements in APD technology have the potential to contribute to the development of compact and portable medical-imaging systems [107,108,109]. The inherent compactness and low power consumption of APDs make them suitable for integration into handheld or wearable imaging devices. Such portable systems could revolutionize point-of-care diagnostics, remote monitoring, and imaging in resource-limited settings [110,111,112]. Future research will focus on miniaturizing APD-based detectors while maintaining their performance characteristics, paving the way for portable medical-imaging solutions that bring imaging capabilities closer to the patient.

Novel device architecture and processing capabilities. Single-photon detector arrays (SiPMs) are compelling candidates for medical imaging, especially in replacing conventional PMTs in positron emission tomography, time-of-flight positron emission tomography, and single-photon emission computed tomography. Their attributes include a high gain with low voltage requirements, fast response times, compactness, and compatibility with magnetic resonance setups.

Since the SiPM output is the summed signal from all the pixels, the detected photon numbers can be identified by screening the amplitude of the output voltage [113]. This makes possible the use of SiPMs as photon-number-resolving (PNR) detectors. Photon-number-resolving detection is a type of photon detection technology that goes beyond the traditional binary detection of whether a photon is present or not. Instead, it provides information about the actual number of photons detected in a given measurement. PNR detectors enable both high-sensitivity and high-dynamic-range detection. This ability is particularly important in certain practical scenarios with largely-varied incident powers. To date, numerous methods have been suggested to improve PNR detection performance [114,115]. Typically, PNR capability can be achieved through either a multiplexing architecture involving multiple detectors or the inherent resolving mechanism within a single device. SiPMs emerge as a promising choice for PNR detection owing to their advantages of low power consumption, cost-effectiveness, and compact design [116].

One more promising direction for the silicon-based APDs is computational single-photon imaging, or computational ghost imaging [117]. This is an imaging technique that leverages the principles of quantum optics and computational algorithms to create images using extremely low levels of light, often down to the single-photon level. Unlike traditional imaging methods that rely on intensity measurements, computation single-photon imaging works with the statistical properties of light, exploiting correlations between photons to reconstruct an image. The core of this technique involves detecting individual photons. Instead of directly measuring the intensity of light at each pixel, computation single-photon imaging relies on the correlation between the intensity patterns of light at different points in the imaging system. Correlation measurements allow the reconstruction of an image even when individual photon detections are sparse. The technique exploits both spatial and temporal correlations in the light field. Spatial correlations involve the relationship between different spatial points in the scene, while temporal correlations consider the correlation over time. One specific approach within computation single-photon imaging is ghost imaging [118]. In ghost imaging, two correlated beams of light are used. One beam illuminates the object, and the other is detected without directly interacting with the object. Although the detectors register only the spatial intensity variations in the second beam, the correlations between the two beams enable the reconstruction of the object’s image. Advanced computational algorithms are employed to process the correlation data and reconstruct the final image. These algorithms can handle the inherent noise associated with the low photon count, leading to improved image quality. Computation single-photon imaging finds applications in scenarios with extremely low light conditions, such as remote sensing, imaging through turbid media, and biomedical imaging. It is particularly advantageous in situations where traditional imaging techniques might fail due to low light levels or high background noise. While computation single-photon imaging is a classical imaging technique, there are quantum-enhanced versions that harness quantum properties for further improvements [119]. Quantum entanglement and quantum correlations can be utilized to enhance the sensitivity and resolution of the imaging system. Some techniques are also developed for the visualization and rapid characterization of events at picosecond time scales. Such an ability to record images with extreme temporal resolution is important for fluorescence lifetime imaging, time-of-flight measurements, and the characterization of ultrafast processes [120].

Nevertheless, challenges in using SiPMs in imaging tools persist, such as the need for optimization in larger matrices, signal amplification, and digitization. Depending on the desired operating wavelength, some other material systems (germanium, A3B5 heterostructures, such as InGaAs/InP and InGaAs/InAlAs, and others) may be exploited for the fabrication of SPAD arrays. For example, in optical communication systems and quantum communication applications, the capability to function at low-loss wavelengths of optical fibers, specifically 1310 nm and 1550 nm, is paramount. Similarly, free space and LIDAR applications necessitate operation within this spectral range [4]. To meet these requirements, there is a need for the development of high-speed and highly sensitive single-photon avalanche diode detectors designed for the short-wave infrared range. One possible solution for the silicon detectors is to extend the operation wavelength into the infrared region [121], which is highly attractive in chemical and biomedical imaging. Recently, some techniques utilizing frequency upconversion in non-linear media [122,123] and two-photon absorption in a silicon avalanche photodiodes were proposed [124]. However, it is very hard for silicon-compatible detectors to compete with highly-developed InGaAs APDs in this spectral range. A3B5 APDs are widely used in emerging single-photon counting capabilities in the near-infrared band, which would also benefit the biomedical-imaging applications [125,126].

Integration with artificial intelligence and machine learning. The integration of APDs with artificial intelligence (AI) and machine-learning algorithms holds significant promise for advancing medical imaging [127,128]. APDs can provide high-quality and high-resolution imaging data, which can serve as valuable inputs for AI-based image reconstruction, analysis, and interpretation [129]. By leveraging AI techniques, APD-based imaging systems can benefit from enhanced image-reconstruction algorithms, real-time image processing, and automated image analysis for faster and more accurate diagnoses. Integration with machine-learning models can also contribute to improved image quality, noise reduction, and artifact correction in APD-based medical-imaging systems [130,131].

Expanding applications in theranostics and image-guided interventions. Theranostics, the combination of diagnostics and therapeutics, is an emerging field that aims to integrate diagnostic imaging with targeted therapy [132,133]. This research field involves the use of nanoparticles [134,135,136,137,138] and novel 2D materials [139,140,141,142,143,144,145]. APDs can play a crucial role in theranostics by providing real-time imaging guidance during interventional procedures, such as minimally invasive surgeries or targeted drug delivery. The high responsivity, fast timing resolution, and compatibility with hybrid imaging modalities make APDs well-suited for image-guided interventions. Future advancements in APD technology will enable the development of integrated systems that seamlessly combine imaging, therapy, and monitoring, leading to personalized and targeted treatments with improved outcomes.

In conclusion, the future of avalanche photodiodes in biomedical applications is promising. The utilization of APDs in medical imaging has the potential to significantly enhance the performance and capabilities of various imaging modalities. Advancements in sensitivity, spectral capabilities, compactness, and integration with AI and machine learning will contribute to the continued evolution of APDs in medical applications. By addressing these future perspectives with continued research and development, APDs have the potential to revolutionize medical imaging by enabling improved diagnostic accuracy, targeted therapies, personalized treatments, and enhanced patient care.

## Data Availability

The authors declare that the data supporting the findings of this study are available within the article.
